# Hospitals during economic crisis: a systematic review based on resilience system capacities framework

**DOI:** 10.1186/s12913-022-08316-4

**Published:** 2022-07-30

**Authors:** Zeynab Foroughi, Parvin Ebrahimi, Aidin Aryankhesal, Mohammadreza Maleki, Shahram Yazdani

**Affiliations:** 1 School of Health Management and Information Sciences, Iran University of Medical Sciences, Tehran, Iran; 2 Virtual School of Medical Education and Management, Shahid Beheshti University of Medical Sciences, Tehran, Iran

**Keywords:** Hospital, Economic crisis, Resilience

## Abstract

**Background:**

Hospitals are the biggest users of the health system budgets. Policymakers are interested in improving hospital efficiency while maintaining their performance during the economic crisis. This study aims at analysing the hospitals’ policy solutions during the economic crisis using the resilience system capacities framework.

**Method:**

This study is a systematic review. The search strategy was implemented on the Web of Science, PubMed, Embase, Scopus databases, and Econbiz search portal. Data were extracted and analysed through the comparative table of resilience system capacities framework and the World Health Organization (WHO) health system’s six building blocks (i.e., leadership and governance, service delivery, health workforce, health systems financing, health information systems, and medicines and equipment).

**Findings:**

After the screening, 78 studies across 36 countries were reviewed. The economic crisis and adopted policies had a destructive effect on hospital contribution in achieving Universal Health Coverage (UHC). The short-term absorptive capacity policies were the most frequent policies against the economic crisis. Moreover, the least frequent and most effective policies were adaptive policies. Transformative policies mainly focused on moving from hospital-based to integrated and community-based services. The strength of primary care and community-based services, types and combination of hospital financing systems, hospital performance before the crisis, hospital managers’ competencies, and regional, specialties, and ownership differences between hospitals can affect the nature and success of adopted policies.

**Conclusion:**

The focus of countries on short-term policies and undermining necessary contextual factors, prioritizing efficiency over quality, and ignoring the interrelation of policies compromised hospital contribution in UHC.

**Supplementary Information:**

The online version contains supplementary material available at 10.1186/s12913-022-08316-4.

## Introduction

Various factors can lead to an economic crisis. One of the most prominent of those was the financial crisis of 2007–2008 that affected a large number of developed countries [1]. This crisis began in the US due to excessive leverage and risky investments and spread worldwide [2]. Moreover, the economic crisis can result from wars, sanctions, climate change, or natural disasters. Similarly, the Corona Virus Disease of 2019 (COVID-19) has devastated the global economy [3]. The World Bank forecasts an almost 8% decrease in global growth and an enormous impact on low- and middle-income countries due to this pandemic [4]. Thus, more than ever, policymakers need to use the experience of previous economic crises.

The financial crisis affected the hospitals seriously [5, 6]. Also, hospitals that constitute one-third of health care expenditures are at the forefront of reforms for cost reduction [7]. Hence, the question is how to maintain hospital performance during the economic crisis and enhance hospital resilience against it. The term ‘*resilience*’ has gained popularity in recent years [8]. The concept of resilience generally refers to “the ability of an individual, household, community or an entire system to withstand the negative impact of shocks” (e.g., an economic crisis) [9].

There is no comprehensive, systematic study that analyzes hospital policies and their outcomes in countries that face an economic crisis within the resilience analysis framework. In this regard, Clemens et al. used document analysis to study hospital reforms in the EU member states during the financial crisis [10]. Thomas et al. examined the resilience of the Irish health system against economic crisis [11], and Alameddine et al. applied the Blanchet et al. resilience capacities framework [12] and Organization for Economic Co-operation and Development (OECD’s) resilience systems’ analysis guideline [13] to evaluate the resilience of health systems in Lebanon and Jordan in accommodating Syrian refugees [14].

The present research aims at identifying and analyzing various hospitals' policy solutions in each of the three resilience system capacities, the effects of these policies on hospital contribution to UHC, and the influential contextual factors in their implementation. Given that detailed information on the impact of interventions in the face of the economic crisis was not reported, we tried to reach a general conclusion based on the available qualitative information.

### Theoretical framework

There are several frameworks for system resilience analysis. Some of them focused on resilient system capacities [12, 15]. Based on the definition by Organization for Economic Co-operation and Development (OECD), a resilient system is a system that can absorb and recover from shocks in the short term while positively adapting and transforming its structure in the long term to cope with changes and maintain its optimal performance [13, 16]. Therefore, the policies adopted in the face of crises divide into one of the following three categories:

(i) **Absorptive capacity:** Policies seek continuity and maintenance of the health services level in terms of quality, quantity, and equity using available resources and keeping the current system’s structure and sustainability.
(ii) **Adoptive capacity:** Policies that imply the system flexibility and make gradual changes in characteristics and activities to provide health services with fewer and different resources.
(iii) **Transformative capacity:** Policies make principal reforms in functions and structure of the health system in response to change [12, 13, 16].

## Methods

This study is a systematic review conducted based on Preferred Reporting Standards for Systematic Reviews 2020 (PRISMA 2020) [17]. It has three main sections. First, the study identifies the effects of the economic crisis on hospital contribution to UHC. Second, it analyzes the policies to deal with these effects. Finally, the study recognizes contextual factors that affect achieving hospital resilience against the economic crisis.

### Eligibility criteria

We included studies published in English until March 1, 2022, with any research design (i.e., quantitative, qualitative, and mixed methods) as well as the grey literature such as research reports and dissertations.

Only publications focused on hospitals were included. In addition, studies included that investigated the effects of the economic crisis on hospitals, how hospitals dealt with the crisis and their policies, and the implications.

Studies published in languages other than English, studies that have examined the health system or one of its levels other than the hospital level, and studies that have examined clinical interventions in times of economic crisis were excluded. The reason for excluding studies that examine the entire health system from the screening was the large number of these studies and the time constraints of researchers. However, this limitation was overcome by reviewing comparative studies.

### Information sources

The search strategy was conducted in Web of Science, PubMed, Embase, Scopus databases, and Econbiz search portal. Econbize is the search portal of economic and business literature that is provided by the German National Library of Economics, Leibniz Information Centre for Economics) to detect relevant studies. Moreover, Google Scholar and ProQuest were searched for grey literature, including reports and dissertations. Also, hand searching in related journals and reference checking of relevant studies were conducted.

### Search strategy

Similar studies were used to detect relevant economic crisis keywords. The most specific and sensitive search strategies were selected by applying different search strategies with various combinations of terms in databases. Table 1 provides the PubMed search strategy as an example.

**Table 1:** The PubMed Search strategy and results

Database	Search Strategy	Number of Studies
PubMed	((Hospitals [Mesh] OR hospital[Title/Abstract] OR hospitalization[Title/Abstract] OR hospitals[Title/Abstract])) AND (“Economic Recession”[Mesh] OR “economic sanction” [Title/Abstract] OR “economic shock”[Title/Abstract] OR “economic recession”[Title/Abstract] OR recession[Title/Abstract] OR “economic crisis”[Title/Abstract] OR “financial crisis”[Title/Abstract] OR “fiscal crisis”[Title/Abstract] OR “banking crisis”[Title/Abstract] OR “economic depression”[Title/Abstract] OR “economic hardship”[Title/Abstract] OR “economic insecurity”[Title/Abstract] OR austerity[Title/Abstract] OR “financial constraint”[Title/Abstract] OR “economic downturn”[Title/Abstract] OR “economic change”[Title/Abstract] OR “economic breakdown”[Title/Abstract] OR “economic turmoil”[Title/Abstract] OR “economic stagnation”[Title/Abstract] OR “economic adversity”[Title/Abstract] OR “economic turbulence”[Title/Abstract] OR “macroeconomic fluctuation”[Title/Abstract] OR “economic crises”[Title/Abstract] OR “financial crises”[Title/Abstract] OR “budget scarcity”[Title/Abstract] OR “restricted budget”[Title/Abstract]))	1197

### Selection process

Among retrieved studies, duplicates were removed using the Endnote software. Two researchers performed screenings. In the first stage, titles and abstracts of studies were examined based on their relevance. Then the studies’ full text is explored based on the eligibility criteria. The screening was conducted by two researchers separately, and controversies were discussed.

### Data collection and synthesis methods

Hospital interventions in the face of the economic crisis were identified and analyzed through the comparative table of the resilience system capacities framework plus WHO’s health system building blocks. In this study, leadership and governance policies refer to rule-based policies (i.e., setting hospital policies, strategies, ownership arrangements, decentralization, stakeholder participation, and contextual factors) [18].

The outcome of program actions, influential contextual factors and studies recommendations were reached by content analysis of related data using MAXQDA 10 software.

### Quality appraisal

Quality of studies assessed based on Mixed Methods Appraisal Tool (MMAT), VERSION2018, which is applicable for quality assessment of various methodologies (i.e., studies with qualitative, quantitative, and mixed-method designs) [19, 20]. Two researchers rated studies on a five-point scale: 0, 25, 50, 75, and 100 (highest level of quality). They resolved the disagreements through discussion and the use of a third researcher. Given that the present systematic review was qualitative, we didn’t exclude any study for having a low rating. However, studies with a higher-quality rating received a higher weight in analysis (i.e., any contradicted results of low-quality studies did not consider).

## Results

The extraction table of study is presented in Additional file 1.

### Study selection and characteristics

**Fig. 1:**
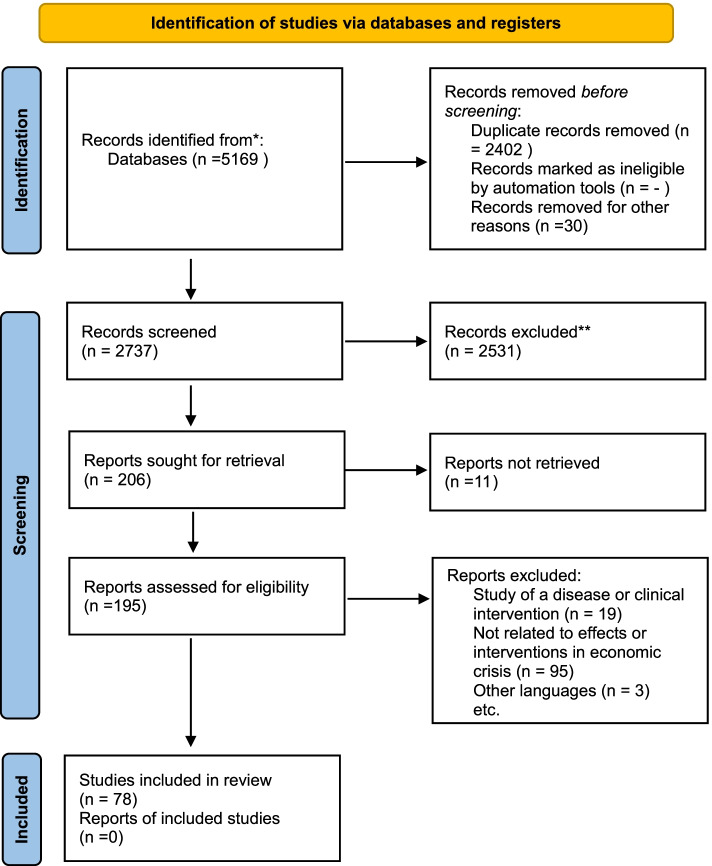
The Prisma Flow diagram of Studies

From a total of 5169 initially detected studies, after screening based on title and abstract,195 studies were examined based on eligibility criteria, of which 78 studies from 36 countries were selected for data extraction (Fig. 1). Among these countries, the highest number of studies belonged to the United States (28%), Greece (17%), Spain (15%) (Table 2).

**Table 2:** Countries and related studies

Country name	References	N of studies
**Austria**	[10, 21]	2
**Brazil**	[22, 23]	2
**Bulgaria**	[10, 24]	2
**Canada**	[25]	1
**China**	[26]	1
**Costa Rica**	[27]	1
**Cuba**	[28],	1
**Cyprus**	[10, 29]	2
**Czech Republic**	[10]	1
**Denmark**	[10]	1
**England**	[10, 30–33],	5
**Estonia**	[10]	1
**Finland**	[34]	1
**France**	[10]	1
**Greece**	[5, 6, 10, 35–45],	14
**Germany**	[46, 47]	2
**Hungary**	[10]	1
**Iran**	[48–59]	11
**Iraq**	[60, 61]	2
**Ireland**	[10, 62, 63]	3
**Italy**	[10, 64],	2
**Latvia**	[10]	1
**Lithuania**	[10]	1
**Luxemburg**	[10]	1
**Malta**	[10]	1
**Netherland**	[10, 30]	2
**Norway**	[30]	1
**Poland**	[10]	1
**Portugal**	[10, 30, 65]	3
**Romania**	[10]	1
**Slovenia**	[10]	1
**Slovakia**	[10]	1
**Spain**	[10, 46, 47, 66–74]	12
**Sweden**	[30]	1
**Tanzania**	[75]	1
**United States**	[76–96]	22

Most of the studies (58%) were non-randomized or descriptive quantitative studies. Moreover, 24% of the studies were non-empirical, and one study was from grey literature.

### Quality appraisal results

Additional file 1 present The quality appraisal results. Almost 58% of the studies had a high rate (100 or 75), and 9% had a medium rate, while only 4% of studies were in a low rating.

### Impacts of economic crisis and adopted policies on achieving universal health coverage at hospital level

As the final goal of a resilient health system is to achieve UHC [97, 98], the impact of economic crisis and adopted policies were reviewed based on their contribution to UHC objectives, including equity in service use, quality, and financial protection [99].

#### Equity in service use

Economic crisis, decrease in household income, and insurance loss reduced hospital services utilization in several countries (Spain, Germany, Iran) [46, 47, 53–57]. Also, in the United States, preventable hospitalization decreased in vulnerable groups [24, 82]. Variously, it caused an increase in emergency, complicated and acute care utilization, especially in vulnerable groups (e.g., Spain and Finland) [34, 67]. Consequently, the inequity in access to hospital services increased (Spain, Germany) [24, 46, 47, 82], which highlights the impact of the crisis on the underserved population’s access to quality primary care [86].

#### Quality

During the economic crisis, hospitals focused on cost reduction and related structural reforms and neglected quality-related issues [30]. For example, the shortage of resources has led to a decline in evidence-based practice in hospitals [38, 75]. In addition, understaffing has led to poor quality and safety of care, patient dissatisfaction [38, 43, 48, 52], and a higher mortality rate [90], which is related to increasing staff workload and decreasing adequate skilled staff [23, 73].

Furthermore, the patient’s waiting time has increased and led to fatal delays and poor quality of care due to the lack of infrastructure, equipment, and medical supplies [28, 37, 59, 61, 75, 90]. For instance, the long waiting lists for surgeries increased patient dissatisfaction threefold in Southern Spain [67].

#### Financial protection

The economic crisis has reduced the available hospitals’ funds, including reimbursement rates, donations, and income (Greece and the US) [35, 79, 80]. Moreover, hospitals’ patient care losses increased due to the reduction in ambulatory care and elective surgeries [29, 60, 79, 85]. Furthermore income from non-patient care activities decreased after the economic crisis [79, 84]. All these factors increased out-of-pocket and informal payments. Policies and measures such as hospitals closure, admission of fewer patients with acute conditions by hospitals, limiting drug prescription, and patients being forced to supply consumables and drugs from outside the hospital have led to an indirect increase in patients’ financial burden [25, 26, 81].

### The frequency of adopted policies based on health system building blocks and resilience capacities

**Fig. 2:**
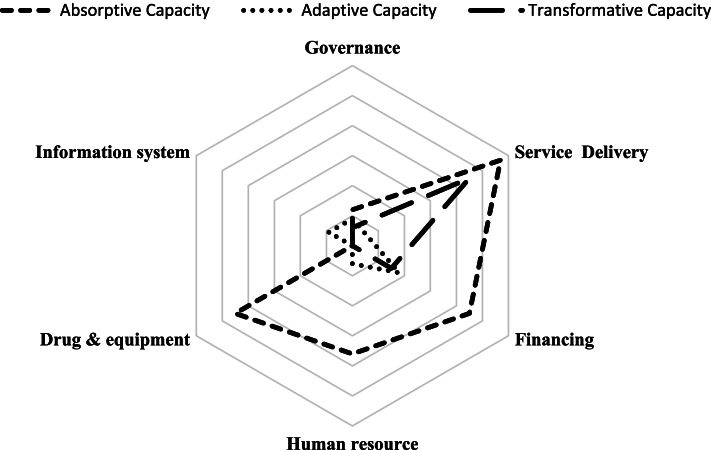
Frequency of hospital policies based on health system six building blocks and resilience capacities

As shown in Fig. 2, absorptive capacity policies are the most frequent policies. Most absorptive capacity policies involve service delivery (57% of countries have implemented such policies). Adaptive capacity policies are the least frequent ones that focus on financing policies (18%). Also, transformative capacity policies are used in service delivery reforms (45%).

### Hospital policies against the economic crisis

The following section refers to various policies of countries in a comparative table of the resilience system capacities framework and the WHO health system’s six building blocks.

#### Absorptive capacity policies

Policies adopted to mitigate the impact of the economic crisis on the essential activities and critical hospital functions (absorptive capacity policies) have aimed to reduce inputs and investment in the health workforce, service delivery, financial resources, and drugs and medical equipment.

Generally, examining the impact and outcomes of these policies indicates a decline in quality, access, equity in service delivery, and greater reliance on informal and Out-Of-Pocket Payments (OOP) [37, 42, 63, 80].

Forty-five percent of countries adopted absorptive financing policies. Most of these countries adopted policies to decrease hospital budgets and payment rates. These policies contributed to an increase in OOP [34]. Only 21% of countries focused on reducing hospital expenditures and investments to save hospital funds (Table 3).

**Table 3:** Absorptive hospital financing policies against the economic crisis

Theme	Sub-themes	Measures	Countries
Reducing Hospitals Funds	Reducing hospital budget and government spending	Budget reduction	Czech Republic [10], Slovenia [10], Austria [10], United States [77], Greece [39], Finland [34]
	Change in the way of budget allocation	Establishing a Unified Yearly Global Budget for all hospitals	Greece [45]
		Upper limits for budgets allocation	China [26]
	Decreasing payment rates of payment systems	Reducing DRG prices	France [10]
		Upper limits for insurance reimbursements	China [26]
		Lowered tariffs paid to providers	Estonia [10], Ireland [10], Romania [10], Slovenia [10], England [31]
		Direct reduction in growth rate of payment	Luxemburg [10], Netherlands [10]
Saving Hospital Funds	Controlling expenditures	Controlling main costs groups in hospitals	Bulgaria [24], Greece [35, 39], England [30], Portugal [30]
	Controlling hospitals investments	Reduction and cancelation of hospital investments/ constructions	United States [79, 85, 93], Romania [10], Spain [10], UK [10],
		Value based purchasing	United States [85]
		Joint purchasing	Greece [45]

In this area, the researchers recommended adopting cost reduction policies in line with utilization [70]. In this regard, about hospital financing policies, some countries have made conscious cost reduction decisions. For example, the UK, Portugal, and Bulgaria adopted policies to reduce main cost groups such as consumables and administrative expenses [24, 30, 35, 39]. Similarly, a Greek study reports an increase in operating expenses following the economic crisis (i.e., overheads, consumables, and security costs [35]. In addition, in Spain, the main reason for increasing costs during the economic crisis was attributed to increased utilization, including hospitalization, surgical admissions, outpatient day-case surgeries, and less to the increase in quasi-prices, including average length of stay, staff-to-bed ratio, and hospital teaching capacity [23, 68, 69].

Concerning the *health workforce*, absorptive policies included:

- Reducing personnel payments (United States, UK, Slovenia, Denmark, Cyprus, Greece, Ireland, Romania, Spain, and Portugal) [5, 6, 10, 35, 77, 94],
- Reducing the number of staff through hiring freezes, layoffs, reduction in staffing positions, reduction in the rate of replacement, and redeploying human resources (United States, Greece, Spain, Denmark, France, Portugal) [5, 6, 10, 27, 39, 71, 78, 79, 85].
- Employing less skilled or unskilled personnel (Netherlands, UK, Slovakia, United States) [5, 6, 10, 35].

These policies have led to a decline in the quantity and quality of hospital staff [37, 42, 63, 80]. In this regard, to mitigate the impact of austerity policies, studies recommended an increase in the human resources quality, improvement of working conditions, and employee motivation [26, 28, 35, 43, 68, 69, 80]. For example, in Spain, reducing the staff wages and salaries and increasing taxes and working hours have reported no negative impact on clinical personnel because of autonomy in the workplace and other working conditions [68, 69]. Also, the results of a US study indicated that highly distressed organizations could serve to motivate employees through group dynamics [91].

Most countries adopted explicit and implicit rationing in service delivery policies. For example, increasing waiting time, introducing or increasing co-payments, reducing patient access, and increasing out-of-pocket payments [34, 35, 41, 75]. However, the results of some studies showed the effectiveness of these policies in countries that strengthened other service delivery methods, including primary care and community-based facilities. For instance, introducing co-payments for emergency department visits in Cyprus reduced avoidable admissions but did not affect unavoidable emergency admissions. Strengthening primary care and community-based facilities in this country was the main reason [50]. Table 4 provides the other absorptive service delivery policies.

**Table 4:** Absorptive Service delivery policies against the economic crisis

Health system building block	Themes	Sub-themes	Countries and their frequency
Service Delivery	Explicit Rationing	Exclusion of some hospital services from basic package	Netherland [10], Denmark [10]
	Implicit Rationing	Increasing waiting time	Estonia [10], Denmark [10], United States [80] Costa Rica [27], England [32]
		Increasing or introducing co-payments	Cyprus [29], Czech Republic, France, Ireland, Romania, Netherlands, and Italy [10]
	Increase revenue-generating services	Increasing ambulatory services	United States [87]
		Change the use of hospital beds (from not-profitable to profitable)	United States [93, 94],
	Reducing Services Costs	Reducing and managing patients Length of Stay	Cuba [28], China [26]
		Decreasing compliance with service standards	United States [80]

Absorptive drugs and medical equipment policies are in two category of supply-side and demand-side policies. About the supply-side policies, some countries adopted policies to reduce drugs price.

Austria, Spain, and the Ireland reduced the price through negotiations and long-term contracts [10]. Also, Belgium introduced a new reimbursement mechanism [10]. Additionally, Malta and Slovakia determine reference price systems for the price reduction [10]. Also, Belgium introduced a new reimbursement mechanism [10]. Additionally, Malta and Slovakia determine reference price systems for the price reduction [10]. Further, countries reduced the use of brand-name drugs and increased the use of generic drugs (Greece, France, Hungary, and the Czech Republic) [10, 39, 44, 80]. Moreover, Greece applied international E-auction [39], and the Czech Republic introduced the auction for drugs and equipment procurement [10].

Demand-side policies concentrated on Reduction in prescription prices (Portugal, Slovenia, Catalonia, Greece, Spain, and Poland) [26, 39, 45], reducing the use of drugs (United States) [80], and Postpone & Reduction of investments for new technologies (United States, Spain) [71, 80].

A limited number of countries implemented absorptive *governance and leadership* policies. These countries focused on changing the behavior of service providers and patients by applying incentives and regulations (Cuba, United States) [28, 77]. Also, the stakeholders’ support is gained by involving them in decision-making (United States, Norway, Portugal) [30, 88].

#### Adaptive capacity policies

In general, adaptive capacity policies included strengthening and adjusting infrastructure through medium-term changes and contributed to the maintenance and improvement of hospital performance in different building blocks. Meanwhile, a Greek study showed that almost all productivity gains were due to technology changes, including applying new methodologies, procedures, and techniques. Thus, studies recommended the utilization and promotion of organizational and managerial reforms [44].

Regarding Governance and Leadership, Cuba, Greece, and Denmark have revised and promoted the clinical processes, guidelines, and hospital policies [10, 28, 35, 44].

Indeed, developing and promoting appropriate policies, guidelines, and regulations are necessary for the prevention of corruption and to manage the diagnostic and treatment choices of physicians [26, 41, 85, 89]. A study in Greece showed that the economic crisis could shift the selection of the surgical technique toward less costly protocols [40]. On the other hand, a US study showed that doctors who had a closer tie to the hospital (e.g., employment) increased their treatment intensity, especially for patients with private insurance [92].

Besides, in governance and leadership, studies highlighted the importance of developing mechanisms for controlling and monitoring performance [22, 26, 88, 89], which is highlighted in the policies of Cuba and Tanzania by promoting financial control and departmental and personnel evaluation systems [28, 75].

Regarding service delivery, only two countries had strategies for enhancing the efficiency of service delivery. These policies have been adopted as solutions that led to using less expensive services (Denmark, Cuba) [10, 28].

Adaptive financing policies were introduced in a limited number of countries, focusing on aligning payments to patient outcomes (Table 5). In this domain, only the United States applied revenue-generating policies [82, 83].

**Table 5:** Policies of countries for aligning payments to patient outcomes

Countries	Policies
United States [83]	Denies payment for “never events” that occur during hospital stays.
	Incentives payments to hospital which have acquired standards.
United States [83], Netherland, Italy [10]	Creating pay-for-performance policies.
England [10]	No reimbursement for readmission in emergency department within 30 days.

Only Greece, Cuba, and Tanzania applied policies in the area of Information systems [28, 45, 75]. These policies were creating and improving the quality of monitoring and controlling hospital financial systems.

Human resource policies are limited to Cuba and Costa Rica [27, 28]. These countries improve personnel efficiency through conducting training programs and evaluation systems.

#### Transformative capacity policies

In terms of transformative capacity, countries have used various mechanisms in governance and leadership, health systems financing, and service delivery domains with a specific focus on transforming service delivery.

On this subject, some of the studies’ recommendations were strengthening the primary care system, restructuring emergency units and creating autonomous emergency departments, and integrating care [5, 10, 28, 29, 39, 41, 67].

In this regard, some countries created new governance and leadership structures to coordinate and manage primary care and hospital services (Greece, US) [41, 44, 45, 86]. Also, several countries conducted hospital integration or closure to reduce hospital care utilization (United States, Canada, Greece, Denmark, Spain) [6, 10, 25, 32, 35, 39, 44, 71, 76, 80, 81, 87].

In addition, there is a necessity to rationalize the payment mechanisms to reflect the exact costs of services [10, 26, 41]. For this purpose, Greece, Portugal, Bulgaria, the Czech Republic, and Cyprus have noted reforms in their payment systems and the shifting from retrospective to prospective payment systems and from cost-based to case-based payment systems [10, 39, 44, 45, 65].

However, the policymakers must consider the effects of cost reduction policies on the patients’ psychosocial wellbeing and access. For example, hospital closure can lead to unemployment, loss of physicians, transportation costs, fear of being hospitalized in other cities, and reduced access to services [25, 44, 81, 88].

### Contextual factors that affect on achieving hospital resilience

Underlying factors that affect on hospital resilience to economic crisis can be examined in six categories, including:

A. *Strong primary care and community-based services*
  A study in Cuba showed that a powerful first line and a family physician system could prevent excessive load on hospitals, making them a place only for the service provided to patients with acute diseases at the second line [28]. Also, appropriate primary care facilities in rural regions of Greece led to lower vulnerability in these regions comparing the urban and suburban areas [5, 36]. In the UK, the transfer of outpatient and ambulatory care into community settings slowed down the increase rate in emergency hospital admissions [32]. However, it didn’t show a significant relationship between social care provision and emergency hospital admissions, which was attributed to ineffective community care [33]. Similarly, a US study showed that the establishment of urgent care centers outside of the hospitals reduced the patients access to Medicare and Medicaid, increased geographical inequalities, and led to the growth of the for-profit health sector due to the lack of proper regulations [81].
B. *Hospital financing system*
  Access to different payment plans [76, 81, 82], lower dependence on non-patient care income, lower need for charity and community-based care, membership of for-profit hospitals in hospital systems, and having a supportive network [76, 95] can make hospitals more resistant against the economic shocks. Contrary, relying on weak insurance funds and the inflexibility of service tariffs has increased the vulnerability of hospitals to the economic crisis [58].
C. *Hospital performance before the crisis*
  Hospitals with lower levels of efficiency before the economic crisis [21] and larger hospitals [39] have achieved more in terms of efficiency and productivity by applying relevant policies. On the other hand, patient-related outcomes (patient safety indicators) in hospitals with better financial performance before the economic crisis have improved post-crisis due to the financial constraints effects [92].
D. *Hospital’s ownership and region*
  After the economic crisis, public and private hospitals have not responded differently to budget constraints [21]. Also, in Greece, the effects of economic crisis were more significant on urban and suburban hospitals comparing rural hospitals [5, 36]. In the same vein, a study in the US showed that being private or public, is located in urban or rural areas, and profitability and financial performance affect a hospital’s quality of care during an economic crisis [80]. In Iran, public hospitals were less vulnerable to the economic crisis [58]. In addition, a study in Spain revealed that public-private partnerships are more resilient against the economic crisis than public and private hospitals [74].
E. *Hospital management competencies*
  Hospital management’s competence in addressing internal and external demands and the information and communication network within hospitals affect their response to the economic crisis [75]. Studies referred to the capacity to manage actors and network interconnectedness as the main factor for improving resilience [23].
F. *Hospital specialties*
  Differences among hospital departments and specialties also moderate the impact of the economic crisis. For example, evidence shows that the economic crisis increased cardiovascular and psychosomatic diseases and suicide attempts while reducing pregnancy and fetal health [36, 39, 67]. In addition, personnel in different departments have shown different levels of vulnerability. For example, a study in Italy found that surgery ward personnel are more at risk of exhibiting symptoms of depression and burnout compared to laboratory personnel [64].

## Discussion

Hospitals are consumers of the majority of health system resources. Therefore, policymakers are interested to know how to deal with hospital policies during an economic crisis. The economic crisis can be a window of opportunity to improve hospitals’ performance in different countries by making resilience. The resilience term raises in the face of various crises and shocks. It is the system viability at the same or a higher level as before the shock. Strategies used in response to a shock can be absorptive (buffering the system from shocks with little or no change in structure), adaptive (limited adjustments in the system structure or processes), or transformative (significant functional or structural change) [100].

**Fig. 3:**
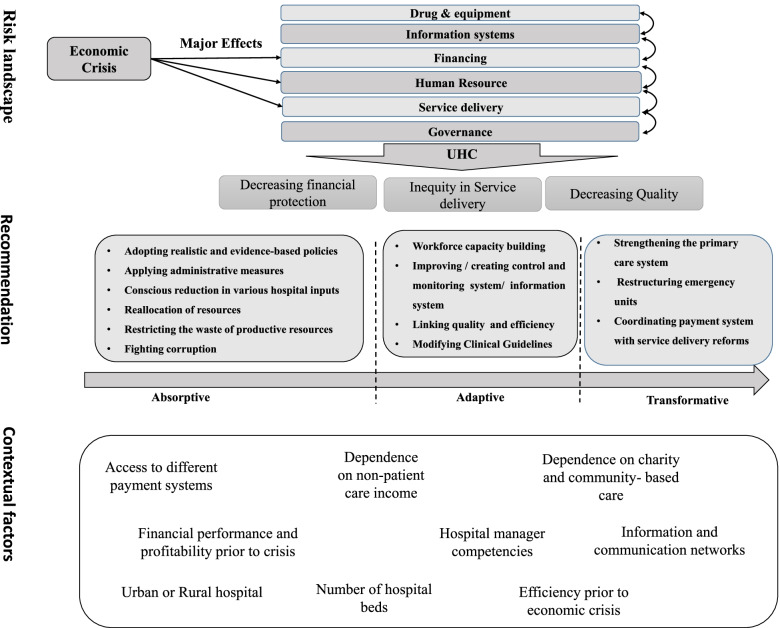
The summary of study results

The present study sought to identify different countries’ solutions in the face of economic crisis and analyze them within the resilience system capacities framework. For this purpose, the impacts of the economic crisis and adopted policies were examined to identify measures for enhancing hospital resilience against the economic crisis and provide insights and directions for health policymakers. Figure 3 summarizes the study findings and recommendations to improve hospital resilience in the economic crisis.

Studies indicated the focus of hospitals on short-term, absorptive measures. As such, failure to identify the root cause of increased hospital costs and failure to make evidence-based cost reduction decisions compromised service quality, access, and fair financial contribution. Moreover, the least frequent policies were adaptive policies, which could play the most significant role in improving hospital performance.

A few countries began reforms in service delivery by moving toward the provision of integrated care and reducing the delivery of hospital services while developing primary care and community-based outpatient care.

Considering the interrelations and the mutual effect of different policies on each other as well as relevant contextual factors is necessary when analyzing the results of this study.

Regarding policies’ interrelations, it is noteworthy that the effects of cost reduction policies on other goals and policies must be considered [101]. A functional cost-containment policy can lead to a decline in the quality of care [26]. For example, hospital restructuring and downsizing policies can create poor working conditions and generate mistrust in the personnel. Personnel dissatisfaction will lead to the provision of low-quality care [37, 42, 64, 68, 77, 78].

Concerning the effect of contextual factors, in the present study, 28% of the reviewed studies are related to the US, which is a federal system where a significant part of the health system follows federal rules and is administered by the private sector with very little public sector participation [102]. Moreover, 17% of the articles are related to Greece, with a highly centralized health system comprising both the public (the National Health System) and the private sector [103]. For instance, a decrease in elective and preventable emergency care services has been observed in countries with a national health system and social insurance such as (e.g., the UK) [10, 27, 29, 32]. On the contrary, various states across the US declined non-profit services and emergency care with low reimbursement [80, 93, 94]. In response to the economic crisis, some countries such as the US resort to public-private partnerships (PPPs) [87], while others such as the UK choose to terminate PPPs contracts.

In addition, the economic crisis and the policies adopted to counter it have had different impacts on different countries. For example, US studies have noted a reduction in efficiency [79, 82], while Greek studies have reported increasing efficiency [35, 39, 44]. This may be due to differences in their financial resource and revenue generation mechanisms since US hospitals are more dependent on non-patient care revenues.

The present study also showed that most countries adopted short-term absorptive policies. These results are in accordance with the study of Clemens et al. that indicates the tendency of countries to make short-term and quick changes to reduce costs, a small number of studies have mentioned structural reforms and integration of care in response to the economic crisis [104]. This may be because most of them are developed countries with appropriate health service infrastructures and less need for structural reforms. For example, implementing the policy of ambulatory and avoidable emergency service reductions requires a powerful and effective primary health care system and community-based services; otherwise, it will lead to increased inequity and reduce patients’ access.

Generally, countries have strived to reduce costs and increase efficiency in each of the three resilience capacities, i.e., absorptive, adaptive, and transformative capacities. However, it has endangered their universal health coverage. Notably, addressing the needs of the covered society and quality care provision takes precedence over financial and cost-saving issues, and improvement of the quality and safety of essential care must continue throughout the economic crisis [26, 38, 39, 65, 95].

In terms of the health system building blocks, the greatest focus was on service delivery and health system financing policies. Also, the governance and leadership and the health information system have the lowest frequency of policies. However, all the health system building blocks are interrelated and should not be considered in isolation [105]. The results of a comparative analysis of hospital reforms in 11 Central and Eastern European Countries between 2008 and 2019 showed that most of these countries had engaged in governance reforms to reduce hospital capacities, purchasing and payment reforms to limit hospital expenditures and to shift service provision to ambulatory care, and all of the countries pursued the objective of reducing the number of hospital beds [104].

The present study has two limitations. The first is in method and inclusion criteria. Studies only were included if they were related to the hospital area. The large number of studies on health systems against the economic crisis and the time constraint of researchers prevented the inclusion of these studies. This could lead to the loss of several hospital-related information in these studies. To overcome this limitation, we included existing comparative studies in this area.

The second limitation concerns the interpretation of the results. Studies didn’t have enough accurate information about the effects of hospital interventions against the economic crisis. Hence, it is not possible to conclude certainly about the impact of the adopted policies. However, we extracted any related data on the effects of policies and concluded based on the frequency of mentioned effects.

Future quantitative and qualitative studies should examine the long-term impacts of any adopted policies on the health system building blocks separately. In this case, a reasonable number of studies can be included in the study. Also, the impacts of each policy will be determined with more confidence.

## Conclusion

The present study was designed to analyze hospital resilience in economic crisis using the resilience system capacities framework. It is hoped that this study could provide a holistic view for policymakers in different countries to choose appropriate policies recognizing different policy options (including absorptive, adaptive, and transformative policies) in each health system six building blocks, and probable consequences on hospital resilience considering contextual factors.

The analysis indicated the importance of considering contextual factors as well as the health system maturity in choosing appropriate policies to improve hospital resilience against the economic crisis. Also, it is necessary to consider the interrelation of the health system’s six building blocks and the effects of policies on each other.

In other words, the focus of countries on short-term absorptive measures without considering related influential contextual factors leads to jeopardizing the UHC. This study highlighted the effects of the strength of primary care and community-based services, hospital managers’ competencies, types and combinations of hospital financing, hospital performance before the crisis, and regional, specialties, and ownership differences between hospitals can affect the effectiveness of adopted policies.

Overall, the study showed that a resilient hospital against the economic crisis is a hospital that has policies to reduce costs and increase efficiency whiteout disturbing the hospital performance in three main building blocks (service delivery, financing, and human resources).

## Supplementary Information

**Additional file 1.**

## Data Availability

All data generated or analysed during this study are included in this published article [and its supplementary information files].

## Abbreviations

COVID-19: Corona Virus Disease of 2019
OECD: Organization for Economic Co-operation and Development
WHO: World Health Organization
UHC: Universal Health Coverage
OOP: Out of Pocket payment
