# Supplementary material for: Hospitals during economic crisis: a systematic review based on resilience system capacities framework
Source: BMC Health Serv Res. 2022 Jul 30;22:977. doi: 10.1186/s12913-022-08316-4 (PMC9339182; doi:10.1186/s12913-022-08316-4)
Supplement: Supplementary file 1 — Additional file 1. [file 12913_2022_8316_MOESM1_ESM.docx]

| **First author, publication year** | **Title** | **Study design** | **Quality Score** | **Main conclusion** |
| --- | --- | --- | --- | --- |
| (Burke et al., 2015) | Economic austerity and healthcare restructuring: correlates and consequences of nursing job insecurity | Quantitative nonrandomized | 75 | **Absorptive capacity**   - Determining incentives for non-profit Hospitals - Creating better competitive condition for public hospitals - Decreasing hospital budget - Hiring freeze - Not filling job vacancies - Restriction in overtime - Reduce growth rate of wage   **Impacts of Economic Crisis**   - Reductions in the number of available hospital staff - Poor working conditions - Endanger the mental well-being of health workforce |
| (De Vos et al., 2010) | Public hospital management in times of crisis: lessons learned from Cienfuegos, | Qualitative | 75 | **Absorptive capacity**   - Increasing public conscience about importance of primary care and family physicians - Reduction of length of stay in hospital services through avoiding unnecessary waiting times   **Adaptive capacity**   - Revision strategies of service delivery in disaster to increase efficiency of emergency room - Improving ‘guidelines to clinical practice’ - creating internal financial control system - Developing an instrument for evaluation at departments level & individual level - improvement of the resolution capacity through a more efficient case management - Developing clinical guideline using Japanese “quality circle” managerial technique - Introducing an effective triage system to improve emergency care - Running an internal financial control system for monitoring of efficiency and budgeting - Restructuring medical staff in order to reduce costs - Implementing staff evaluations and rating them based on these evaluations - Improving quality of data through digitalization of data collection and fast track- analysis   **Transformative Capacity**   - Creating an integrated public health system to diminish pressure on the emergency services   **Impacts of Economic Crisis**   - Increase in the volume of patients and admission to emergency and ambulatory care - Increased hospital efficiency |
| (Bledsoe et al., 2012) | Human capital and process improvement | Non empirical | - | **Absorptive capacity**   - Creating Steering committee   **Recommendation**   - importance of preparation and involvement of internal and external stakeholders |
| (Burnett et al., 2016) | Using institutional theory to analyse hospital responses to external demands for finance and quality in five European countries. Journal of health services research & policy | Qualitative | 100 | **Absorptive capacity**   - Creating cost control mechanism for three main costs groups in hospitals   **Impacts of Economic Crisis**   - Divert of hospital attention from quality-related issues   **Recommendation**   - Gain support from internal & external stakeholders |
| (Clemens et al., 2014) | European hospital reforms in times of crisis: Aligning cost containment needs with plans for structural redesign? | Comparative study | _ | **Absorptive capacity**   - Exclusion of some hospital services from basic package - Reducing or removing reimbursement for elective services - Increasing waiting time - Co-payments increased or introduced - Reducing hospital beds - Decreasing hospital budget - Lowered tariffs paid to providers - DRG prices reduced - Direct reduction in growth rate of payment - Reduce, abandon and cancellation of investments/ capital projects/ technologies upgrade - Reduction of the number of health professionals through redundancies - Employing less skilled The or un-skilled personnel - Cutting salaries - Renegotiation of contracts for reduction in pharmaceutical and medical product price - Reducing prescription price - Lowering the price of medical goods by new reimbursement mechanism - Procurement of medical equipment and drugs by Auction - Increasing use of generic drugs   **Adaptive capacity**   - Improving ‘guidelines to clinical practice’ - Introducing alternative and less expensive intervention in clinical guideline - Creating pay-for-performance policies - No reimbursement for readmission in emergency department within 30 days   **Transformative Capacity**   - Hospital closure or Merger - hospital department closure - Stop plans to build hospitals - Move towards non‐hospital setting - Redirecting funds or restructuring hospitals toward promoting outpatient and day care services - Integrating service providers - Changing the use of hospital beds to residential care - Centralization of emergency and specialized services - Promoting or implementing prospective payment system like DRG |
| (Dong, 2015) | Performing well in financial management and quality of care: evidence from hospital process measures for treatment of cardiovascular disease | Quantitative nonrandomized | 100 | **Absorptive capacity**   - Increasing waiting time for specialized and private/non- emergency/elective services - Reducing hospital beds - Decreasing compliance with service standards - Reduction in staffing - Postpone & Reduction of investments for new technologies - Reduce use of drugs   **Transformative Capacity**   - Hospital closure or Merger |
| (Boddiger, 2012) | Costa Rica restructures health system to curb financial crisis | Non empirical | - | **Absorptive capacity**   - Increasing waiting time for specialized and private/non- emergency/elective services - Increasing surgeries without increasing staff   **Transformative Capacity**   - Restructuring medical staff in order to reduce costs |
| (Lewis, 2019) | More reform of the English National Health Service: from competition back to planning? | Non empirical | - | **Absorptive capacity**   - Increasing waiting time for specialized and private/non- emergency/elective services - provision of emergency and ambulatory care outside of hospitals   **Transformative Capacity**   - Using multidisciplinary community providers to shift outpatient and ambulatory care into community settings. - focus on reduce spending on hospital care and to use enhanced primary care - New care models to deliver more integrated care - Moving from competition to collaboration in NHS |
| (Petrou and Ingleby, 2019) | Co-payments for emergency department visits: a quasi-experimental study | Quantitative nonrandomized | 100 | **Absorptive capacity**   - Introducing copayment to reduce avoidable ED |
| (Choi, 2017) | Hospital Capital Investment During the Great Recession | Quantitative nonrandomized | 100 | **Absorptive capacity**   - Decreasing low-reimbursing / non-profitable while making beds available for higher-paying elective hospitalizations - Reduce, abandon and cancellation of investments/ capital projects/ technologies upgrade   **Impacts of Economic Crisis**  an increase in the volume of patients and admission to emergency and ambulatory care departments in public hospitals |
| (Sussman et al., 2010) | Hospitals during recession and recovery: vulnerable institutions and quality at risk |  |  | **Absorptive capacity**   - Made an agreement with employees to take large cuts in pay and vacation time to prevent laying off 10% of the staff - Decreasing low-reimbursing / non-profitable while making beds available for higher-paying elective hospitalizations |
| (Berliner, 2004) | The Crisis of the Los Angeles County Public Hospital System: A Harbinger for the Nation | Non empirical  (report) | - | **Absorptive capacity**   - Increasing ambulatory services - Reducing hospital beds   **Adaptive capacity**   - downsized bed capacity, expanded ambulatory services - Establish public private partnership for expanding ambulatory care program, private ambulatory clinics   **Impacts of economic crisis**   - An increase in the volume of patients and admission to emergency and ambulatory care departments |
| (Kalafati, 2012) | How Greek healthcare services are affected by the Euro crisis. Emergency Nurse | Non empirical | _ | **Absorptive capacity**   - Reducing hospital beds - Hiring freeze - Reduction in staffing - Decrease in staffing positions - Increasing age of retirement - Increasing civil servant tax - Cutting salaries   **Transformative Capacity**   - Hospital closure or Merger |
| (Polyzos et al., 2013) | Reforming reimbursement of public hospitals in Greece during the economic crisis: implementation of a DRG system | Non empirical | - | **Absorptive capacity**   - Establishing a Unified Yearly Global Budget for all hospitals - Joint purchasing of medical services and goods to achieve expenditure reductions through price-volume agreements   Concrete pharmaceutical policies (i.e., negative or positive lists according, electronic prescriptions, etc.) /Reintroduce positive list  **Adaptive capacity**   - creating operational web-based facility to collect updated and reliable data and improve the capability to monitor the economical operation of the hospitals and to make performance comparisons - Improving hospital infrastructure and technology in order to cost reduction - unification of financial sources   **Transformative Capacity**   - Establishing a unified social security fund which is responsible for coordinating primary care, regulate contracting with healthcare providers , monitoring and set quality and efficiency standards - Promoting or implementing prospective payment system like DRG |
| (Yan et al., 2019) | China’s new policy for healthcare cost-control based on global budget: a survey of 110 clinicians in hospitals | Quantitative nonrandomized | 100 | **Absorptive capacity**   - Limiting the duration of hospitalization, average cost in hospitalization, usage of top-ranked drugs, and usage of examination/drug/surgery - Defining upper limits for budgets allocation and insurance reimbursements - Restricting average drug prescription costs in outpatients - Regularly rank and limit the use of top-ranked drugs such as antibiotics and inhibiting the behaviors of excessive medical treatments   **Impacts of Economic Crisis**  increase in the number of hospital visits |
| (Dong, 2015) | Performing well in financial management and quality of care: evidence from hospital process measures for treatment of cardiovascular disease | Qualitative | 100 | **Absorptive capacity**   - Decreasing compliance with service standards - Reducing hospital beds - Increasing waiting time - Reduction in staffing - Postpone & Reduction of investments for new technologies - Reduce use of drugs   **Transformative Capacity**   - Hospital closure or Merger   **Contextual factors**  Being private or public, being located in urban or rural areas, and profitability and financial performance affect a hospital’s quality of care during economic crisis.  **Impact of economic crisis**   - reductions in the number of available hospital staff - less skilled staff - reduced the funds available to hospitals - reduction in the profit margins of private hospitals |
| (Mitropoulos et al., 2018) | The impact of economic crisis on the Greek hospitals' productivity | Quantitative nonrandomized | 100 | **Absorptive capacity**   - Creating cost control mechanism for three main costs groups in hospitals (hospital supplies, administration costs…) - Redeploy human resources - Implementing international e-auction for procurements - Increasing use of generic drugs - Concrete pharmaceutical policies (i.e. negative or positive lists according, electronic prescriptions, etc.) /Reintroduce positive list   **Adaptive capacity**   - Decreasing cost in-efficiency of reimbursement policies / Rationalize payment methods - Increasing economic of scale   **Transformative Capacity**   - Hospital closure or Merger - Promoting or implementing prospective payment system like DRG   **Contextual factors**   - larger hospitals have achieved more in terms of efficiency |
| (Keskimäki, 2003) | How did Finland's economic recession in the early 1990s affect socio-economic equity in the use of hospital care? | Quantitative nonrandomized | 100 | **Absorptive capacity**   - Government reduced spending and increased out of pocket - increase in the volume of patients and admission to emergency and ambulatory care departments   **Impact of economic crisis**   - reduction in the average length of stay of patients - hospital utilization among deprived groups increased - an increase in households’ out-of-pocket payments |
| (Hellowell et al., 2019) | Austerity and Hospitals in Deficit: Is PPP Termination the Answer? | Quantitative descriptive | 50 | **Absorptive capacity**  Lowered tariffs paid to providers  Early termination of PPP contracts to cost reduction  **Transformative Capacity**  Payments made under Public Private Partnerships are ring-fenced and indexed to inflation |
| (Burke et al., 2011) | Hospital restructuring and downsizing: Effects on nursing staff well-being and perceived hospital functioning | Quantitative nonrandomized | 75 | **Absorptive capacity**   - laying off workers - Hiring freeze   **Adaptive capacity**   - rearranges jobs, units and reporting relationships   **Impact of economic crisis**   - reductions in the number of available hospital staff - leaving the profession by nurses due to poor working conditions - endanger the mental well-being of health workforce |
| (Berger et al., 2020) | Determinants of soft budget constraints: How public debt affects hospital performance in Austria | Quantitative nonrandomized | 50 | **Contextual factors**   - hospitals with lower levels of efficiency prior to the economic crisis achieved more in terms of efficiency and productivity by applying relevant policies - public and private hospitals have not responded differently to budget constraints |
| (Martins et al., 2019) | Access and effectiveness inpatient care indicators and economic crisis: analysis based on the Brazilian Unified Health System data | Quantitative descriptive | 75 | **Impact of economic crisis**   - reduced access and increased out-of-pocket payment in poor people |
| (Vaseva et al., 2016) | Outcomes analysis of hospital management model in restricted budget conditions. Biotechnology & Biotechnological Equipment | Quantitative nonrandomized | 75 | **Absorptive capacity**   - Creating cost control mechanism for three main costs groups in hospitals (hospital supplies, administration costs)   **Impact of economic crisis**   - preventable hospitalization decreased in vulnerable groups |
| (James, 1999) | Closing rural hospitals in Saskatchewan: on the road to wellness? Social science & medicine | Qualitative | 75 | **Transformative Capacity**   - Hospital closure or Merger |
| (Seamer et al., 2019) | Did government spending cuts to social care for older people lead to an increase in emergency hospital admissions? An ecological study | Quantitative nonrandomized | 100 | **Recommendation**   - No significant relationship between social care provision and emergency hospital admissions, which was attributed to ineffective community care |
| (Kaitelidou et al., 2016) | The impact of economic crisis to hospital sector and the efficiency of Greek public hospitals | Quantitative nonrandomized | 75 | **Absorptive capacity**   - Creating cost control mechanism for three main costs groups in hospitals (hospital supplies, administration costs) - Cutting salaries - Reforms in hospital policies and procedures:   **Adaptive capacity**   - Reforms in hospital policies and procedures:   **Transformative Capacity**   - Integration and merger of public hospitals and dividing them into groups with common management |
| (Gkentzi et al., 2019) | Economic recession and attendances in the Pediatric Emergency Department. BioMed research international | Quantitative nonrandomized | 50 | **Contextual factors**   - urban and suburban hospitals have been affected more significantly by economic crisis compared to rural hospitals |
| (Karamanoli, 2015) | 5 years of austerity takes its toll on Greek health care | Non empirical | - | **Impact of economic crisis**   - Aging of public hospital personnel due to migration of young physicians and nurses |
| (Keramidou and Triantafyllopoulos, 2018) | The impact of the financial crisis and austerity policies on the service quality of public hospitals in Greece | Quantitative descriptive | 100 | **Impact of economic crisis**   - increased physical and psychological strain on clinical personnel - decline in evidence-based practice in hospitals - poor quality and safety of care - patient dissatisfaction |
| (Koutserimpas et al., 2019) | The burden on public emergency departments during the economic crisis years in Greece: a two-center comparative study | Quantitative nonrandomized | 75 | **Absorptive capacity**   - Reduction in staffing - Increasing age of retirement - Cutting salaries   **Transformative Capacity**   - Horizontal integration between hospitals and vertical integration of hospitals   **Contextual factors**  urban and suburban hospitals have been affected more significantly by economic crisis compared to rural hospitals |
| (Schizas et al., 2019) | The profile of patients receiving public and private surgical services in Greece during the economic crisis: a comparative study | Quantitative descriptive | 50 | **Impact of economic crisis**  economic crisis can shift the choice of the surgical technique toward less costly protocols |
| (Souliotis et al., 2016) | Informal payments in the Greek health sector amid the financial crisis: old habits die last | Quantitative descriptive | 50 | **Impact of economic crisis**   - Increase in households’ out-of-pocket payments - Increase in under-the-table payments to physicians and nurses |
| (Spiridakis et al., 2015) | Impact of financial crisis on the workload, training, personal and social life of the residents in surgical specialties in Greece | Quantitative descriptive | 25 | **Impact of economic crisis**   - Devotion of less time by clinical staff to training and personal development due to high workload and mental and physical fatigue - Increase in the volume of patients and admission to emergency and ambulatory care departments - Endanger the mental well-being of health workforce - Increase in demand for acute care, urgent cases, complicated pregnancies and cardiovascular diseases, especially in vulnerable groups |
| (Stavrianou et al., 2018) | Informal Caregivers in Greek Hospitals: a Unique Phenomenon of a Health System in Financial Crisis | Quantitative nonrandomized | 100 | **Impact of economic crisis**   - Reductions in the number of available hospital staff following economic crisis - Poor quality and safety of care, patient dissatisfaction |
| (Xenos et al., 2017) | Efficiency and productivity assessment of public hospitals in Greece during the crisis period 2009–2012 | Quantitative nonrandomized | 75 | **Absorptive capacity**   - Reduction in the use of brand name drugs   **Adaptive capacity**   - Reforms in hospital policies and procedures - Improving hospital infrastructure and technology in order to cost reduction - Establishing a unified social security fund   **Transformative Capacity**   - Integration and merger of public hospitals and dividing them into groups with common management - Promoting or implementing prospective payment system like DRG |
| (Kiernan, 2019) | Public policy failure in healthcare: The effect of salary reduction for new entrant consultants on recruitment in public hospitals | Non empirical | - | **Impact of economic crisis**   - Inability to recruit and retain personnel - Leaving the profession by nurses due to poor working conditions |
| (Carr and Beck, 2019) | Clinician responses to management control: Case evidence from a university hospital during the fiscal crisis | Qualitative | 100 | **Impact of economic crisis**   - lack of participation by clinicians in the development of cost containment policies, lack of management accountability vis-à-vis clinicians, and poor management control mechanisms have led to unaccountability on the part of clinicians - endanger the mental well-being of health workforce - an increase in the volume of patients and admission to emergency and ambulatory care |
| (Carta et al., 2017) | Risk for depression, burnout and low quality of life among personnel of a university hospital in Italy is a consequence of the impact one economic crisis in the welfare system? | Quantitative nonrandomized | 50 | **Impact of economic crisis**   - endanger the mental well-being of health workforce |
| (Abásolo et al., 2017) | Financial crisis and income-related inequalities in the universal provision of a public service: the case of healthcare in Spain | Quantitative nonrandomized | 100 | **Impact of economic crisis**  people are less likely to utilize preventive care |
| (Córdoba-Doña et al., 2018) | Withstanding austerity: Equity in health services utilisation in the first stage of the economic recession in Southern Spain | Quantitative nonrandomized | 75 | **Impact of economic crisis**   - Reductions in the number of available hospital staff - Increase in patient dissatisfaction - Increase in the volume of patients and admission to emergency and ambulatory care departments - Hospital utilization among deprived groups increased |
| (Esteban‐Sepúlveda et al., 2019) | The nurse work environment in Spanish nurses following an economic recession: From 2009 to 2014 | Quantitative nonrandomized | 100 | **Transformative Capacity**   - Hospital closure or Merger   **Impact of economic crisis**   - Staff reduction in hospitals - Inability to recruit and retain personnel |
| (Fontova-Almató et al., 2020) | Evolution of Job Satisfaction and Burnout Levels of Emergency Department Professionals during a Period of Economic Recession. | Quantitative nonrandomized | 100 | **Impact of economic crisis**  reductions in the number of available hospital staff |
| (Bernal-Delgado et al., 2020) | Factors underlying the growth of hospital expenditure in Spain in a period of unexpected economic shocks: A dynamic analysis on administrative data | Quantitative descriptive | 100 | **Impact of economic crisis**   - Increase in costs during economic crisis more to increased utilization, including hospitalization, surgical admissions, outpatient day-case surgeries, and less to the increase in quasi-prices, including average length of stay, staff-to-bed ratio, and hospital teaching capacity |
| (Borra et al., 2019) | Austerity, healthcare provision, and health outcomes in Spain | Quantitative descriptive | 100 | **Absorptive capacity**   - Reduction in rate of replacement of retired workers - Postpone & Reduction of investments for new technologies - hospital department closure |
| (Strong, 2017) | Working in scarcity: Effects on social interactions and biomedical care in a Tanzanian hospital | Qualitative | 100 | **Adaptive capacity**   - Creating a cash collection office and computerized accounting system to keep track of user fees - Creating a system to prevent financial mismanagements including a computerized accounting system and a cash collection office   **Impact of economic crisis**   - Decline in evidence-based practice in hospitals - Fatal delays in service delivery - Higher number of urgent stays compared to elective stays, and an increase in demand for acute care, urgent cases, complicated pregnancies and cardiovascular diseases, especially in vulnerable groups - Reduced access and increased out-of-pocket payment in poor people - Increase in under-the-table payments to physicians and nurses |
| (Bazzoli et al., 2014) | Hospital financial performance in the recent recession and implications for institutions that remain financially weak | Quantitative nonrandomized | 100 | - **Transformative Capacity** - Hospital closure or Merger - **Contextual factors** - access to different payment plans |
| (Chen et al., 2019) | How the great recession affects performance: a case of Pennsylvania hospitals using DEA | Quantitative nonrandomized | 100 | **Absorptive capacity**   - Reduction and cancelation of hospital construction plans - laying off workers   **Impact of economic crisis**   - Reductions in the number of available hospital staff - Decline in hospital efficiency during economic crisis - Economic crisis has reduced the funds available to hospitals including, reimbursement rate, donations to hospitals, and hospital income - Patient care losses due to the reduction in ambulatory care and elective surgeries |
| (Henry, 2015) | Hospital closures: The sociospatial restructuring of labor and health care | Qualitative | 75 | **Absorptive capacity**  Employing less skilled The or un-skilled personnel  **Transformative capacity**  Hospital closure or Merger  Move towards non‐hospital setting  **Contextual factors**  Closing hospitals and creating urgent care center (UCC)  access to different payment plans |
| (Izon and Pardini, 2015) | A Stochastic Frontier Analysis of California Safety-Net Hospital Cost Inefficiency through the Great Recession | Quantitative nonrandomized | 100 | **Adaptive capacity**   - Decreasing cost in-efficiency of reimbursement policies / Rationalize payment methods - Applying retail strategies : using new technology and updating facilities to expand profitable services and attracting insured patients / Cream-skimming : allocation of resource to lower-cost services   **Impact of economic crisis**   - people are less likely to utilize preventive care - the demand for acute care and costly processes with small profit margins increases in the short-term - changes in patient composition - decline in hospital efficiency during economic crisis   **Contextual factors**   - access to different payment plans - volume of patients and admission to emergency and ambulatory care departments in public hospitals - private hospitals are faced with a lower volume of patients and |
| (Izón and Pardini, 2017) | Cost inefficiency under financial strain: a stochastic frontier analysis of hospitals in Washington State through the Great Recession | Quantitative nonrandomized | 100 | **Adaptive capacity**   - Denies payment for “never events” that occur during hospital stays - Incentives payments to hospital which have measures of hospital-acquired conditions legislation - Creating pay-for-performance policies - Applying retail strategies : using new technology and updating facilities to expand profitable services and attracting insured patients / Cream-skimming : allocation of resource to lower-cost services |
| (Schuhmann, 2010) | Can net income from non-patient-care activities continue to save hospitals? The times when hospitals could rely on non-patient-care activities to offset losses on patient care may be over | Quantitative descriptive | 75 | **Impact of economic crisis**   - Income from non-patient care activities decreases after economic crisis |
| (Shortt, 2014) | A historical perspective of the effect of the great recession on hospitals | Non empirical (Report ) | - | **Absorptive capacity**   - Reduction and cancelation of hospital construction plans - Reduce, abandon and cancellation of investments/ capital projects/ technologies upgrade - Purchasing capital investment based on their return /Purchasing equipment based on their value to patients - laying off workers - Decrease in staffing positions   **Impact of economic crisis**   - Higher number of urgent stays compared to elective stays, and an increase in demand for acute care, urgent cases, complicated pregnancies and cardiovascular diseases, especially in vulnerable groups - Hospitals are faced with increasing patient care losses due to the reduction in ambulatory care and elective surgeries |
| (White et al., 2018) | The effect of the global financial crisis on preventable hospitalizations among the homeless in New York State | Quantitative nonrandomized | 75 | **Adaptive capacity**   - Establishing High-Risk Pool Program for the uninsured with preexisting health conditions that provided complete primary care and outpatient substance abuse treatment   **Impact of economic crisis**   - higher number of urgent stays compared to elective stays, and an increase in demand for acute care, urgent cases, complicated pregnancies and cardiovascular diseases, especially in vulnerable groups - During economic crisis, homeless people, the uninsured, and other communities have had a significantly higher rate of outpatient admissions, which highlights the impact of crisis on underserved population’s access to quality primary care |
| (Stewart and Smith, 2011) | An Examination of Contemporary Financing Practices and the Global Financial Crisis on Nonprofit Multi-Hospital Health Systems | Mixed method | 25 | **Transformative capacity**   - Many community hospitals and academic medical centers sought the simultaneous pursuit of vertical and horizontal integration   **Impact of economic crisis**   - Economic crisis led to an increase in interest costs, which is one of the main sources of income for non-profit hospitals, leaving them with losses from the early retirement of their variable rate debt and reduced liquidity |
| (Lee, 2015) | The Impact of Financial Crisis on the Financial Asset in California Hospitals | Quantitative nonrandomized | 100 | **Absorptive capacity**   - Hospital closure or Merger   **Impact of economic crisis**   - Increasing mortality rate |
| (Boakye et al., 2020) | The impact of interpersonal support, supervisory support, and employee engagement on employee turnover intentions: Differences between financially distressed and highly financially distressed hospitals | Quantitative nonrandomized | 75 | **Impact of economic crisis**   - Migration of specialists to the private sector |
| (Adelino et al., 2019) | Hospital financial health and clinical choices: evidence from the financial crisis | Quantitative nonrandomized | 100 | **Contextual factors**   - Patient-related outcomes (patient safety indicators)in hospitals with better financial performance before the economic crisis, have improved post-crisis due to the effect of financial constraints |
| (Sussman et al., 2010) | Hospitals during recession and recovery: vulnerable institutions and quality at risk | Non empirical | - | **Absorptive capacity**   - Decreasing low-reimbursing / non-profitable while making beds available for higher-paying elective hospitalizations - Made an agreement with employees to take large cuts in pay and vacation time to prevent laying off 10% of the staff   **Adaptive capacity**   - Creation of mechanisms for linking quality and efficiency |
| (Sánchez‐Recio et al., 2021) | Inequities in hospitalisation in a South European country: Lessons learned from the last European recession | Quantitative nonrandomized | 100 | **Impacts of Economic crisis**   - Decrease in hospitalization - Increase inequity in hospitalization |
| (Lostao et al., 2017) | Socioeconomic position and health services use in Germany and Spain during the Great Recession | Quantitative nonrandomized | 100 | **Impacts of Economic crisis**   - No change in accessibility to health system |
| (Moreno et al., 2021) | Trends and equity in the use of health services in Spain and Germany around austerity in Europe | Quantitative nonrandomized | 50 | **Impacts of Economic crisis**   - No socioeconomic differences in service use |
| (Manzano García et al., 2021) | Does the economic crisis contribute to the burnout and engagement of Spanish nurses? | Quantitative descriptive | 100 | **Absorptive capacity**   - Decrease in recruitment rate of health professionals - freezing of posts - Prioritization in financing - Reduce investment in infrastructures   **Adaptive capacity**   - Introducing provincial cost containment committee (PCCC) to review ad monitor any expenditure   **Impacts of Economic crisis**   - Deterioration of health professional’s psychosocial well being and burnout of human resources   **Contextual factor**   - Hospital management capacity - The quality of actor networks |
| (Fana and Goudge, 2021) | Austerity, resilience and the management of actors in public hospitals: a qualitative study from South Africa | Qualitative | 100 | **Decrease in quality of care because of low skill management and staff, lack of medical supplies, and increasing** medical negligence   - Increase in wages - Increase in medical products costs |
| (Nasiri et al., 2021) | The economic effect of Covid-19 on hospital industry in Iran and the worled | Non empirical | - | **Contextual factors**   - Being public or private hospital - Financing mechanism |
| (Ortega-Díaz et al., 2020) | Multilevel Analysis of the Relationship between Ownership Structure and Technical Efficiency Frontier in the Spanish National Health System Hospitals | Quantitative nonrandomized | 100 | **Contextual factors**   - Public-private partnerships are more successful in economic crisis than public or private hospitals |
| (Fingar et al., 2018) | Shifts in Medicaid and Uninsured Payer Mix at Safety-Net and Non-Safety-Net Hospitals During the Great Recession | Quantitative nonrandomized | 75 | **Contextual factors**   - Financing mechanisms |
| (Gorji, 2014) | Sanctions against Iran: The Impact on Health Services | Non empirical | - | **Impacts of Economic crisis**   - Decreasing access to essential medical products - Interruption and delay in service delivery of hospitals |
| (Murphy et al., 2020) | Economic sanctions and Iran’s capacity to respond to COVID-19 | Non empirical | - | **Impacts of Economic crisis**   - Decreasing access to essential medical products |
| (Shahabi et al., 2020). | Physical rehabilitation in Iran after international sanctions: explored findings from a qualitative study | Qualitative | 100 | **Impacts of Economic crisis**  Decreasing quality of drugs and hospital equipment  Increase in service delivery expenditures |
| (Tabrizi, 2019) | Impact of economic sanctions on orthopedic residency program (education & training) | Non empirical | - | **Impacts of Economic crisis**  Increase in service delivery expenditures |
| (Rad et al., 2017) | Does Economic Instability Affect Healthcare Provision? Evidence Based on the Urban Family Physician Program in Iran | Quantitative nonrandomized | 75 | **Impacts of Economic crisis**  Decrease in service utilization |
| (Danaei et al., 2019) | The harsh effects of sanctions on Iranian health | Non empirical | - | **Impacts of Economic crisis**  Decrease in service utilization |
| , (Yazdi-Feyzabadi et al., 2020), | The Health Consequences of Economic Sanctions: Call for Health Diplomacy and International Collaboration | Non empirical | - | **Impacts of Economic crisis**   - Decrease in service utilization |
| (Aloosh et al., 2019), | Economic sanctions threaten population health: the case of Iran | Qualitative | 25 | **Impacts of Economic crisis**   - Decrease in service utilization |
| (Kokabisaghi et al., 2019) | Impact of United States political sanctions on international collaborations and research in Iran | Non empirical | - | **Impacts of Economic crisis**   - Decrease in service utilization |
| (Garfield et al., 2003) | Health Care in Iraq | Non empirical | - | **Impacts of Economic crisis**   - Increase in number of cases and reduce health workers |
| (Rawaf, 2005) | The health crisis in Iraq | Non empirical | - | **Impacts of Economic crisis**   - Decrease in outpatient services - Increase in poor quality private hospital services |
| (Craig and Koleda, 1978) | The Urban Fiscal Crisis in the United States, National Health Insurance, and Municipal Hospitals | Non empirical | - | **Transformative capacity**   - Municipal hospital closure - Changing the ownership of government operating hospitals to medical schools operating hospitals   **Contextual factors**   - The role of national health insurance programs will decrease by restrictive eligibility criteria and lees innovative reimbursement systems |

CRAIG, J. & KOLEDA, M. 1978. The Urban Fiscal Crisis in the United States, National Health Insurance, and Municipal Hospitals. *International Journal of Health Services,* 8**,** 329-349.
